# Optimizing surgical strategies for subaxial cervical fracture-dislocation: a facet and disc injury-based approach

**DOI:** 10.3389/fsurg.2025.1718134

**Published:** 2025-11-26

**Authors:** Shiyong Wang, Rubin Yao, Xiangdong Gong, Xin Miao, Yandong Chu, Kaishun Yang, Zhaohui Ge

**Affiliations:** 1 Department of Orthopedics, General Hospital of Ningxia Medical University, Yinchuan, China; 2 The First Clinical Medical College, Ningxia Medical University, Yinchuan, China; 3 Department of Spinal Surgery, First Affiliated Hospital of Dali University, Dali, Yunnan, China

**Keywords:** subaxial cervical, fracture-dislocation, facet joint injury, disc injury, surgical approach

## Abstract

**Objective:**

To investigate the influence of facet joint and intervertebral disc injuries on surgical approach selection in subaxial cervical fracture-dislocation.

**Methods:**

We retrospectively analyzed 150 patients with subaxial cervical fracture-dislocations, stratified by surgical approach: anterior (anterior-only or anterior-posterior) and posterior (posterior-only or posterior-anterior). Preoperative computed tomography (CT) and magnetic resonance imaging (MRI) were assessed to identify injury patterns, and radiographic and clinical outcomes were compared before and after surgery.

**Results:**

The mean patient age was 48.84 ± 11.68 years, and 86.7% completed at least 12 months of follow-up. The anterior group exhibited significantly higher rates of unilateral facet injuries (53.5% vs. 18.4%), perched/separated facets (59.4% vs. 34.7%), F2-type fractures (46.5% vs. 2%), and disc extrusion (40.9% vs. 15.4%). In contrast, the posterior group had more locked facets (55.1% vs. 15.8%) and non-fracture dislocations (79.6% vs. 23.8%). Bilateral injuries were commonly associated with locked or separated facets and absence of fracture. Patients undergoing combined posterior-anterior surgery had greater incidence of bilateral facet involvement and disc protrusion. Spinal alignment significantly improved postoperatively in all groups (*P* < 0.05), with no statistical difference in alignment correction between approaches. Twenty-four patients showed no neurological recovery, and 20 patients died during the follow-up period. Complication rates were higher in the posterior group (14.3%) compared to the anterior group (2.97%).

**Conclusion:**

Facet and disc injury patterns strongly affect surgical decision-making. Anterior approaches are preferable for disc extrusion or facet fractures, while posterior surgery is suited for locked facets without fractures. Accurate injury classification via imaging can guide individualized treatment and improve outcomes.

## 1 Introduction

Subaxial cervical fracture-dislocation is a severe spinal injury, commonly involving unilateral (51.2%) or bilateral (48.8%) facet dislocations, with the C6/C7 level most frequently affected (38.5%) (1) . Associated bilateral facet injuries, spinal canal stenosis, and cord compression often imply neurological compromise (1). In neurologically intact patients, closed reduction combined with cervical collar immobilization may be attempted, in contrast, neurological deficits may compromise the success rate of closed reduction (2). However, due to extensive capsuloligamentous disruption, post-reduction instability is common, making surgical intervention the preferred option (3, 4) .

Anterior approaches are widely used, offering effective decompression, realignment, restoration of disc height, and preservation of lordosis (5–8). Heavy-weight skull traction combined with anterior surgery can effectively treat severe subaxial cervical dislocations, achieving complete decompression, good reduction, restoration of intervertebral height, and maintenance of cervical lordosis (9, 10); Anterior discectomy can also facilitate reduction, and in cases of difficult reduction, partial vertebral resection may be employed, followed by additional posterior decompression if necessary, thus minimizing the morbidity of combined approaches (11); Zhang et al. (7) demonstrated that anterior facet joint resection could enhance the efficiency of anterior reductions, potentially avoiding the need for posterior surgery. Nevertheless, these procedures are technically demanding, with risks of neurovascular injury, and anterior fixation alone may be insufficient in bilateral facet dislocations with vertebral translation (12). Such patients often require supplementary posterior stabilization, particularly those with severe neurological deficits. Aman et al. (13) found that bilateral facet joint dislocation and traumatic PLL rupture are independent risk factors for failure of anterior cervical discectomy and fusion (ACDF) in the treatment of cervical spine fractures. A 10-year follow-up study comparing anterior and posterior short-segment fixation found that posterior surgery had fewer complications, including less throat pain, and better cervical disability index scores at final follow-up (14). However, it was associated with longer operative time, increased blood loss, and longer hospital stays (14). A systematic review reported similar overall outcomes between approaches, though surgical failures occurred only in the anterior group (15).

Despite these advances, controversy remains regarding the optimal surgical approach, particularly in complex cases with coexisting disc and facet joint injuries. Clinicians often face challenges in selecting the most appropriate strategy to achieve spinal stability, neurological protection, and functional recovery. Therefore, a better understanding of the correlation between facet joint and disc injury patterns and surgical outcomes is crucial to guide individualized treatment strategies. Preoperative imaging-based stratification of injury severity could assist in predicting reduction feasibility and approach choice. The Spinal Cord Buffer Space (SCBS) concept proposed by Leng et al. (16) suggests that posterior reduction is feasible in SCBS-positive cases, while SCBS-negative status necessitates anterior decompression, emphasizing the decisive impact of disc herniation on surgical approach selection in subaxial cervical dislocation. However, this framework overlooks the equally critical influence of facet joint morphology and injury configuration in surgical decision-making. Additionally, the AO Spine classification (17) does not provide definitive guidance on selecting the surgical approach for the treatment of Type C dislocations. In this context, this study retrospectively analyzes subaxial cervical fracture-dislocation cases to correlate facet and disc injury patterns with surgical outcomes, aiming to improve preoperative decision-making, optimize approach selection, and ultimately enhance clinical outcomes for patients with this devastating injury.

## 2 Methods

### 2.1 General information

This study was approved by the Clinical Research Ethics Committee of the The First Affiliated hospital of Dali University (DFY20230301001). Inclusion criteria were: (1) confirmed cervical spine trauma; (2) radiological evidence of cervical dislocation from our hospital or a referring facility; (3) complete imaging data; (4) surgical treatment performed at our center; and (5) postoperative follow-up of ≥1 year (excluding deceased patients), with follow-up completed by December 2023. Exclusion criteria included: (1) old fracture-dislocations (>3 weeks); (2) pathological fractures; (3) mismatch between diagnostic records and imaging; (4) voluntary discharge or refusal of surgery; and (5) missing key imaging data.

**Figure 1:**
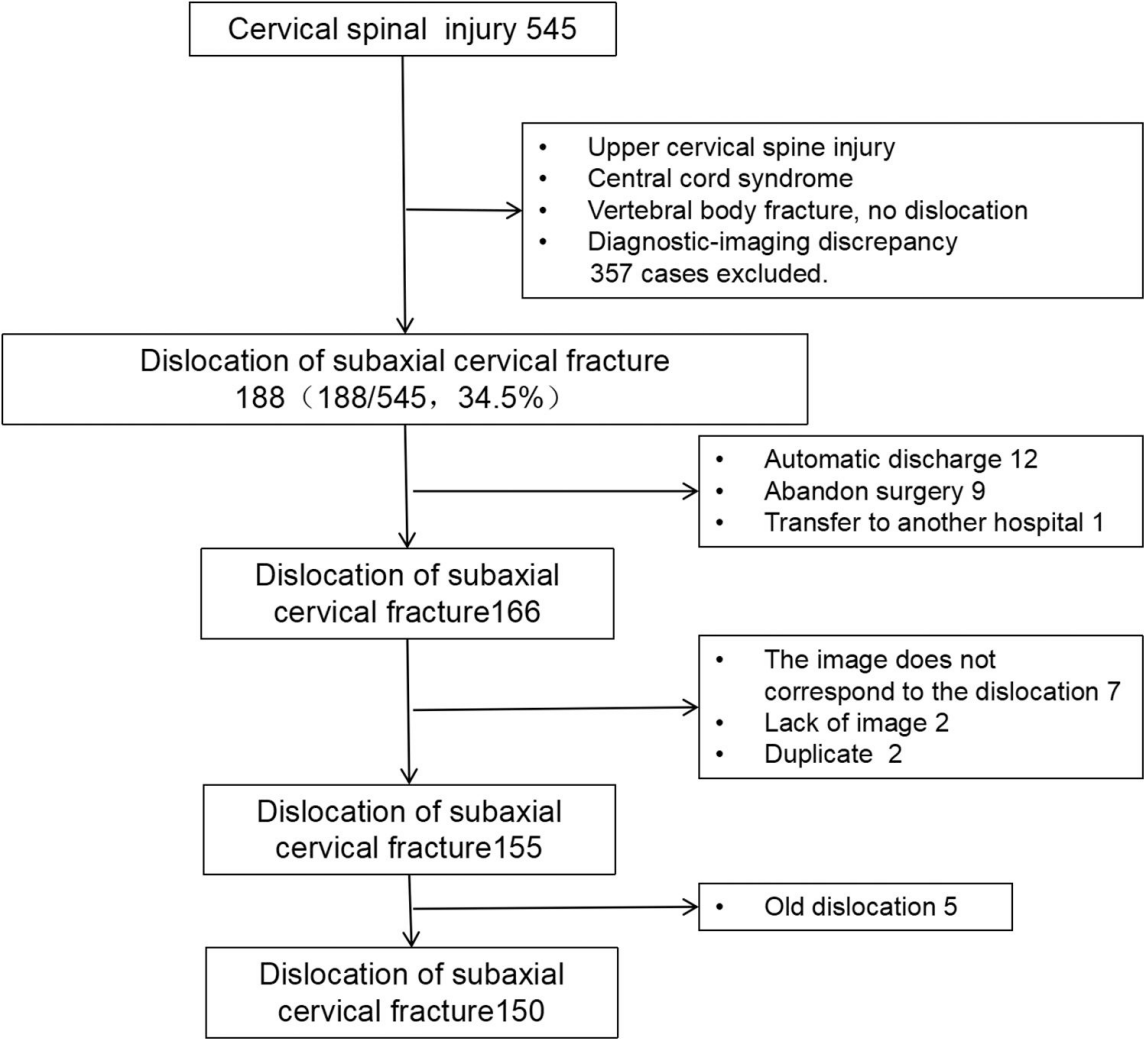
Inclusion criteria for patients with cervical spine fracture and dislocation.

Among 545 patients with cervical spinal trauma admitted between January 2014 and January 2022, 188 (34.5%) were diagnosed with subaxial fracture-dislocations. After applying exclusion criteria, 150 patients were ultimately included (Figure 1).

### 2.2 Classification methods

**Figure 2:**
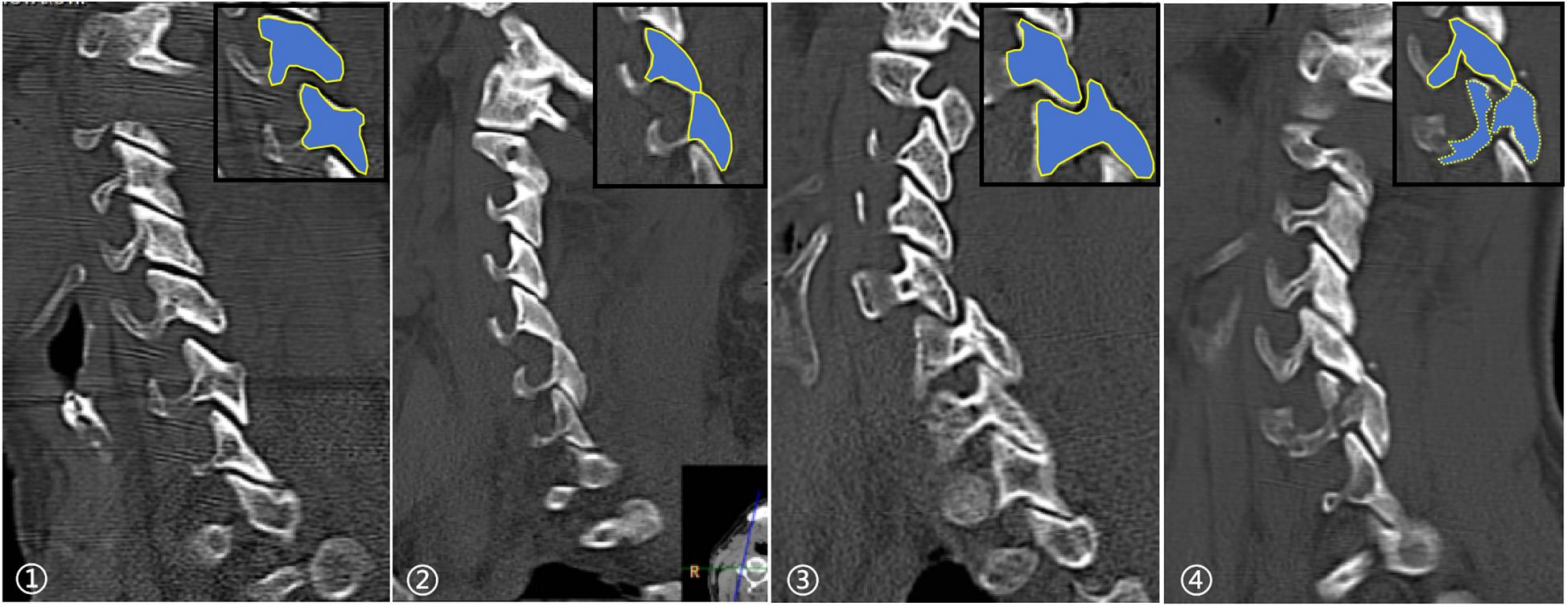
Anatomic alignment of facet joints: (1) separated; (2) perched; (3) locked; (4) facet joints perched with fracture.

**Figure 3:**
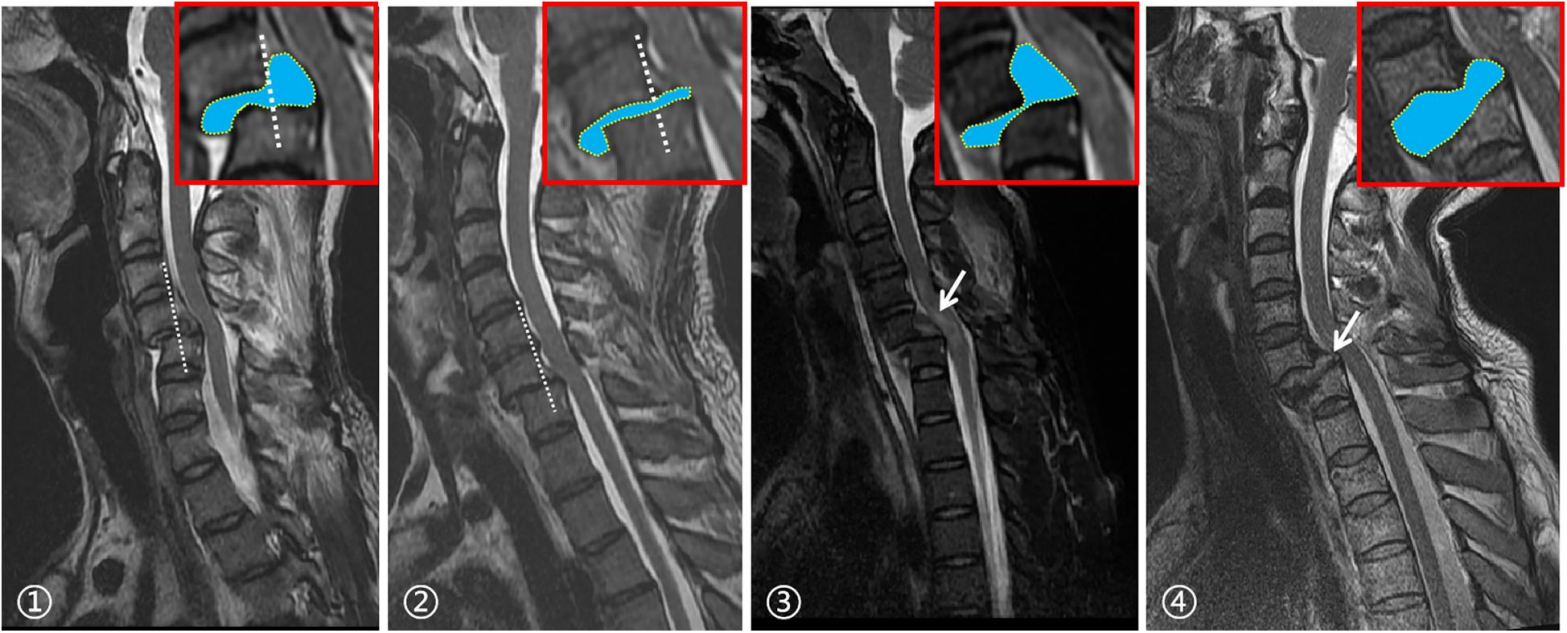
Types of disc injury: (1) traumatic disc herniation: defined on a T2-weighted sagittal midline MRI by drawing a line along the posterior margin of the superior vertebral body. If the disc extends beyond this line posteriorly, it is classified as a traumatic herniation. (2) Non-Herniation: If the disc does not extend past the line, it is considered non-herniated. (3) Disc Extrusion: Disc material seen migrating freely posterior to the vertebral body on sagittal MRI (white arrow) indicates extrusion. (4) Disc Protrusion without Extrusion: Disc bulges without posterior migration beyond the posterior margin of the vertebral body (white arrow), consistent with protrusion without extrusion.

AO Classification of Subaxial Cervical Spine Injury (17): This study included only AO Type C injuries and further subclassified facet joint and disc injuries as follows: (1) Facet Joint Injuries: (1) Unilateral vs. bilateral involvement; (2) Anatomical alignment based on CT sagittal images (Figure 2): normal, separated, perched, and locked. If accompanied by facet fractures, injuries were further classified as F1, F2, or F3. For asymmetric bilateral injuries, the more severe side was recorded (injury severity ranked as: normal < separated < perched < locked; F1 < F2 < F3). (2) Disc Injuries: Assessed on sagittal cervical MRI and categorized as: no protrusion, protrusion, or extrusion (Figure 3). (3) Vertebral Body Fractures: Fractures at the dislocated segments were classified as A1–A4 based on the AO classification. Neurological function: Assessed using the American Spinal Injury Association (ASIA) Impairment Scale (18).

### 2.3 Surgical methods

**Preoperative Traction:** For patients unable to undergo immediate surgery, skull traction was initiated after MRI at 3 kg, gradually increasing to 6 kg. For those undergoing immediate surgery, traction began at 3 kg after general anesthesia, increasing by 2.5 kg every 10–15 min, up to 12 kg, with C-arm fluoroscopy to monitor alignment. In cases with cervical disc extrusion, traction was used solely for stabilization, followed by anterior decompression. If reduction failed, posterior surgery was performed. All patients were intubated with a video laryngoscope or fiberoptic bronchoscope. The operation was performed by four chief physicians.

**Anterior Approach:** The dislocation level was exposed, osteophytes removed, and the annulus fibrosus incised. Distraction was achieved with Caspar screws, and reduction was performed using distraction and levering. Partial vertebral resection was done if necessary. An interbody or titanium cage with autograft/allograft was inserted, followed by anterior plate fixation.

**Posterior Approach:** A midline incision was made, and paraspinal muscles were dissected. Facet reduction was achieved using burring and levering in locked or perched facets. Lateral mass screws and contoured rods were placed.

**Combined Approach:** A combination of anterior-posterior or posterior-anterior procedures was performed, adjusting techniques to ensure optimal spinal realignment.

### 2.4 Postoperative management

Intravenous second-generation cephalosporins were used for infection prophylaxis (clindamycin for allergic patients). Postoperative care included dehydrating agents and neurotrophic drugs for spinal cord or nerve injury. On day one, patients could elevate the bed or sit up with cervical collar fixation. On day two, mild spinal cord injury patients were encouraged to ambulate with collar support. For ASIA A and B patients, air mattresses and pneumatic compression devices were used for pressure ulcer and thrombosis prevention. Urinary catheters were removed on day one (except for ASIA A/B). Drains were removed after 48–72 h, and sutures on day 10–14. Follow-up imaging occurred at 3, 6, and 12 months.

### 2.5 Evaluation for therapeutic effect: radiological and spinal cord injury

**Figure 4:**
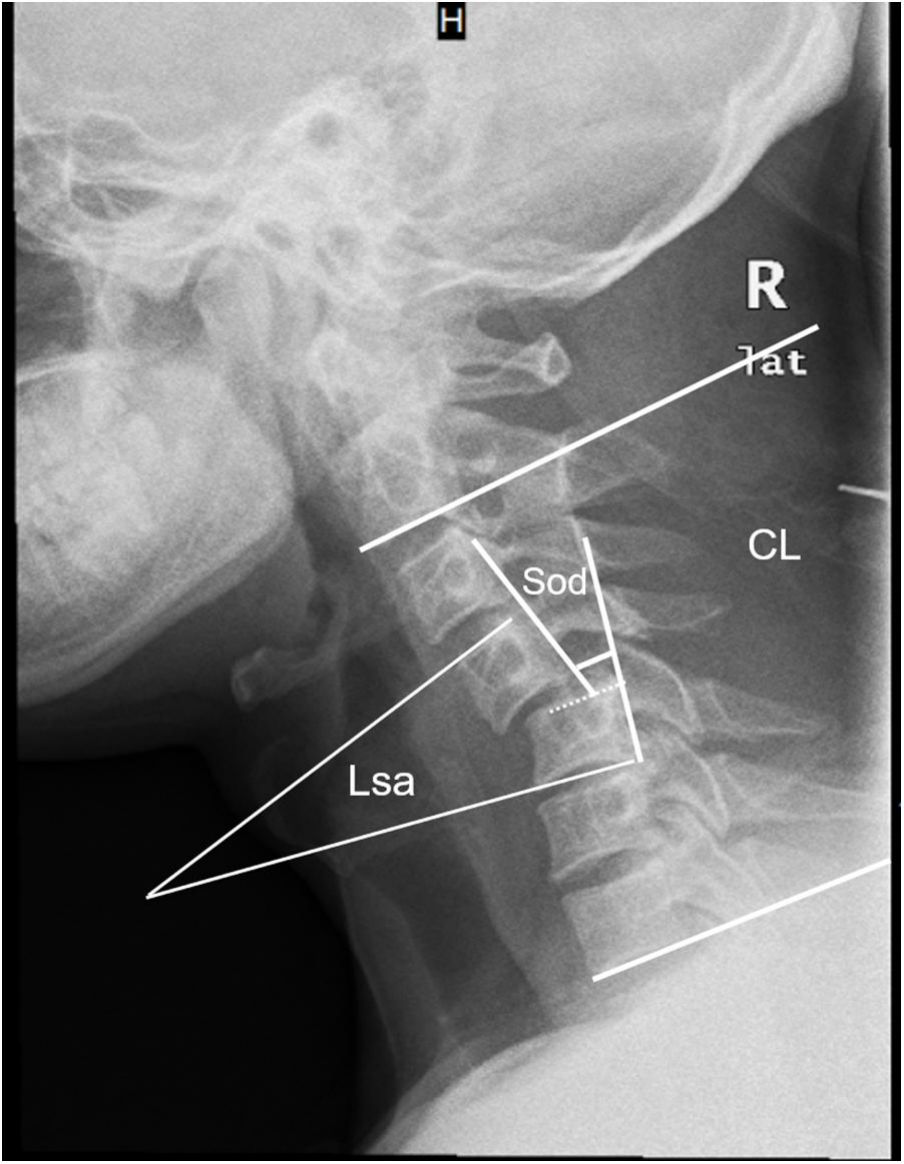
Radiological measurement parameters of the subaxial cervical spine: Lsa: was measured between the upper endplate of the cephalad vertebra and the lower endplate of the caudal vertebra at the dislocated level; CL: was the angle between the lower endplates of C2 and C7; Sod: was the horizontal distance between the posterior vertebral lines of the cephalad and caudal vertebrae.

Alignment was evaluated using three parameters (Figure 4): Segmental Cobb angle (Lsa); Cervical lordosis (CL); Anterior slippage distance (Sod) (7). Measurements were performed using PACS software (Hangzhou Shixuan Co., Ltd.). If x-ray was inconclusive, mid-sagittal CT was used. Fusion was evaluated via CT based on intra-graft and extra-graft bridging bone (InGBB and ExGBB) (19). Neurological status was evaluated using ASIA grade at admission and at final follow-up.

### 2.6 Statistical methods

Statistical analyses were conducted using SPSS 20.0. Continuous variables: expressed as mean ± standard deviation; compared using independent-samples *t*-test or paired *t*-test (for pre/post comparisons). Categorical variables: analyzed using chi-square test or Fisher's exact test (if expected frequency <5 or total *n* < 40). Strength of association: assessed using Cramer's V (for nominal variables) and Gamma (for ordinal variables), ranging from 0 (no correlation) to 1 (perfect correlation). A *p*-value < 0.05 was considered statistically significant.

## 3 Results

### 3.1 Demographic data and surgical-related indicators

**Figure 5:**
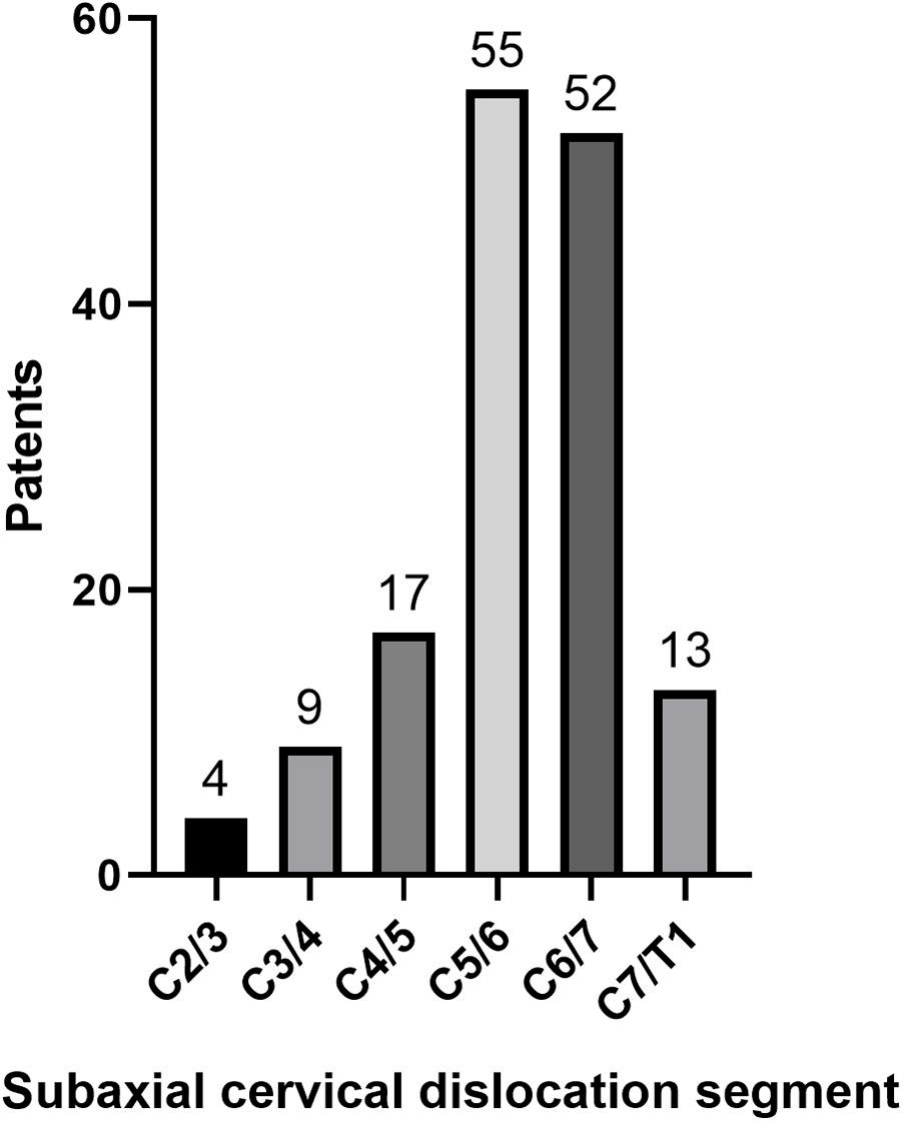
Distribution of dislocation segments in subaxial cervical spine.

A total of 150 patients met the inclusion criteria (118 males, 32 females; mean age 48.84 ± 11.68 years, range 20–90). The mean time from injury to admission was 43.6 ± 69.52 h (range 2–360), and from admission to surgery was 5.09 ± 2.87 days (range 1–21). Injury mechanisms included falls (106), motor vehicle accidents (32), heavy object impact (5), and others (7). Most injuries involved the C5/6 and C6/7 segments (71.3%, Figure 5). 101 underwent anterior approaches (90 anterior-only, 11 anterior-posterior), and 49 underwent posterior approaches (13 posterior-only, 36 posterior-anterior). The mean follow-up was 23.27 ± 7.92 months (range 1–42). Postoperative CT at 1-year confirmed solid fusion in all surviving patients.

Patients in the posterior group had more severe preoperative neurological impairment (ASIA A/B: 36.7% vs. 11.9%, *p* = 0.003), distinct dislocation patterns (Cramer's V = 0.362, *p* = 0.002), and a higher incidence of injuries at the C2/3 (8.2% vs. 0%) and C7/T1 (18.4% vs. 4%) levels. Posterior procedures were associated with longer operative times (*p* < 0.001), greater blood loss (*p* < 0.001), and longer hospital stays (*p* = 0.009). No significant differences were found in demographics or vertebral fracture rates (*p* > 0.05) (Table 1).

**Table 1:** Demographic and surgical variables of patients in both groups.

Variables		Anterior group	Posterior group	*t/x^2^*	*P*
Age (y)		48.21 ± 12.1	50.14 ± 10.76	−0.951	0.343
Gender, *n* (%)	m/f	79 (78.2)/22 (21.8)	39 (79.6)/10 (20.4)	0.037	0.847
Pre-op ASIA, *n* (%)	A	10 (9.9)	12 (24.5)	−0.447^a^	**0**.**003**^c^
	B	2 (2)	6 (12.2)		
	C	11 (10.9)	5 (10.2)		
	D	57 (56.4)	22 (44.9)		
	E	21 (20.8)	4 (8.2)		
Dislocation segment, *n* (%)	C2/3	0 (0)	4 (8.2)	0.362^b^	**0**.**002**^c^
	C3/4	7 (6.9)	2 (4.1)		
	C4/5	14 (13.9)	3 (6.1)		
	C5/6	41 (40.6)	14 (28.6)		
	C6/7	35 (34.7)	17 (34.7)		
	C7/T1	4 (4)	9 (18.4)		
Vertebral fracture, *n* (%)	yes/no	18 (17.8)/83 (82.2)	13 (26.5)/36 (73.5)	1.526	0.282
Time to Hospital after Injury (h)		40.53 ± 64.88	49.92 ± 78.58	−0.774	0.440
hospitalization to surgery (days)		5.02 ± 2.96	5.22 ± 2.70	−0.422	0.683
Duration of hospitalization (days)		14.98 ± 9.78	20.39 ± 14.82	−2.665	**0**.**009**
Operating time (min)		110.11 ± 50.54	196.39 ± 53.78	−9.602	**<0**.**001**
Intra-op hemorrhage (mL)		81.04 ± 85.71	259.22 ± 196.18	−6.083	**<0**.**001**
Post-op drainage (mL)		52.77 ± 76.04	297.78 ± 236.21	−7.085	**<0**.**001**
No. of fused segments		1.22 ± 0.48	1.51 ± 1.14	−1.724	0.090

m, male; f, female; op, operation; ASIA, American spinal injury association.

Bold indicates *p* < 0.05.

a Effect size analysis: Gamma (ordered variable).

b Effect size analysis: Cramer's V (unordered variable).

c Fisher's exact test.

### 3.2 Types of facet joint and disc injuries

#### 3.2.1 Types of facet joint injuries

Given the complexity of facet joint injuries, this study compared unilateral and bilateral injuries, anatomical alignments, and fracture types to better characterize their features. Perched facets (69.8% vs. 37.9%) and F1/F2 fractures (81.0% vs. 33.3%) were significantly more common in the unilateral injury group, whereas bilateral injuries were more frequently associated with locked facets (40.2%) and non-fracture cases (62.1%) (all P < 0.001, Cramer's V = 0.342–0.494; Table 2). Fracture type was significantly correlated with anatomical alignment (Cramer's V = 0.27, P < 0.001), with F1/F2 fractures predominantly presenting with perched facets (65.6%–72.9%; Table 3). In patients without facet fractures, locked facets were significantly more common (46%; Table 3).

**Table 2:** Comparison of unilateral and bilateral facet joint injury.

	Facet joint injury		*Cramer's V*	*P (Fisher)*
	Unilateral	Bilateral		
Anatomical alignment, *n* (%)				
normal	1 (1.6)	3 (3.4)	0.342	**0**.**001**
separated	10 (15.9)	16 (18.4)		
perched	44 (69.8)	33 (37.9)		
locked	8 (12.7)	35 (40.2)		
Fracture of facet joint, *n* (%)				
no	9 (14.3)	54 (62.1)	0.494	**<0**.**001**
F1	18 (28.6)	14 (16.1)		
F2	33 (52.4)	15 (17.2)		
F3	3 (4.8)	4 (4.6)		

Bold indicates *P* < 0.05.

**Table 3:** Analysis of facet joint fractures and anatomical alignment.

	Fracture of facet joint				*Cramer's V*	*P (Fisher)*
	no	F1	F2	F3		
Anatomical alignment, *n* (%)						
Normal	3 (4.8)	0 (0)	1 (2.1)	0 (0)	0.27	**<0**.**001**
Separated	14 (22.2)	4 (12.5)	5 (10.4)	3 (42.9)		
Perched	17 (27.0)	21 (65.6)	35 (72.9)	4 (57.1)		
Locked	29 (46.0)	7 (21.9)	7 (14.6)	0 (0)		

Bold indicates *P* < 0.05.

#### 3.2.2 Comparison of facet and disc injury in the anterior and posterior groups

Facet joint injuries displayed distinct patterns between surgical approaches. Unilateral facet injuries were predominant in the anterior group (53.5% vs. 18.4%, *p* < 0.001), whereas bilateral injuries were more frequent in the posterior group (81.6% vs. 46.5%). Anatomical alignment also differed markedly (Cramer's V = 0.428, *p* < 0.001): perched facets were mainly observed in anterior cases (59.4% vs. 34.7%), while locked facets predominated in posterior cases (55.1% vs. 15.8%). Facet fracture patterns were strongly correlated with the surgical approach (Cramer's V = 0.559, *p* < 0.001). F2-type fractures were common in the anterior group (46.5% vs. 2%), whereas non-fracture dislocations dominated in the posterior group (79.6% vs. 23.8%). Moreover, traumatic disc extrusion occurred significantly more often in anterior approach cases (40.9% vs. 15.4%, *p* = 0.026). (Table 4).

**Table 4:** Comparison of the type of joint and disc injuries between the two groups.

Operative approach			Anterior group	Posterior group	*x^2^*	*P*
Facet joint injury, *n* (%)		Unilateral	54 (53.5)	9 (18.4)	16.685	**<0**.**001**
		Bilateral	47 (46.5)	40 (81.6)		
Anatomical alignment, *n* (%)		Normal	2 (2)	2 (4.1)	0.428^b^	**<0.001** ^a^
		Separated	23 (22.8)	3 (6.1)		
		Perched	60 (59.4)	17 (34.7)		
		Locked	16 (15.8)	27 (55.1)		
Fracture of facet joint, *n* (%)		No	24 (23.8)	39 (79.6)	0.559^b^	**<0**.**001**^a^
		F1	24 (23.8)	8 (16.3)		
		F2	47 (46.5)	1 (2)		
		F3	6 (5.9)	1 (2)		
Traumatic disc, *n* (%)	Protrusion	Yes	44 (56.4)	26 (53.1)	2.247	0.299
		No	57 (43.6)	23 (46.9)		
	Extrusion^c^	Yes	18 (40.9)	4 (15.4)	4.491	**0**.**026**
		No	26 (59.1)	22 (68.6)		

Bold indicates *P* < 0.05.

a Fisher's exact test.

b Effect size analysis: Cramer's V (unordered variable).

c Extrusion disc: patients with free nucleus pulposus to the posterior border of the vertebral body after traumatic (Figure 3).

### 3.3 Intra-group comparison between anterior subgroup and posterior subgroup

In subgroup analyses, the anterior-posterior group, despite having only 72.7% with disc herniation, still required posterior surgery due to high rates of bilateral facet joint injury (72.7%), facet perched/locked (90.9%), and F0–F1 injuries (81.8%). In the posterior-anterior group, although bilateral facet joint injury (91.7%), facet perched/locked (97.2%), and F0–F1 injuries (94.5%) were more prevalent, 63.9% still needed anterior surgery for disc herniation (Table 5).

**Table 5:** Comparison of facet joint and disc injury types within subgroups.

Operative approach			Anterior group		*x^2^*	*P*	Posterior group		*x^2^*	*P*
			Single anterior	Combined anterior-posterior			Single posterior	Combined posterior-anterior		
Facet joint injury, *n* (%)		Unilateral	51 (56.7)	3 (27.3)	3.404	0.065	6 (46.2)	3 (8.3)	0.431^b^	**0**.**007**^a^
		Bilateral	39 (43.3)	8 (72.7)			7 (53.8)	33 (91.7)		
Anatomical alignment, *n* (%)		Normal	2 (2.2)	0 (0)	0.129^b^	0.633^a^	2 (15.4)	0 (0)	0.45^b^	**0**.**017**^a^
		Separated	22 (24.4)	1 (9.1)			2 (15.4)	1 (2.8)		
		Perched	52 (57.8)	8 (72.7)			2 (15.4)	15 (41.7)		
		Locked	14 (15.6)	2 (18.2)			7 (53.8)	20 (55.6)		
Fracture of facet joint, *n* (%)		No	20 (22.2)	4 (36.4)	0.246^b^	0.096^a^	10 (76.9)	29 (80.6)	0.159^b^	0.821^a^
		F1	19 (21.1)	5 (45.5)			3 (23.1)	5 (13.9)		
		F2	45 (50)	2 (18.2)			0 (0)	1 (2.8)		
		F3	6 (6.7)	0 (0)			0 (0)	1 (2.8)		
Traumatic disc, *n* (%)	Protrusion	Yes	34 (37.8)	8 (72.7)	0.221^b^	**0**.**048**^a^	3 (23.1)	23 (63.9)	6.387	**0**.**011**
		No	56 (62.2)	3 (27.3)			10 (76.9)	13 (36.1)		
	Extrusion^c^	Yes	16 (47.1)	2 (25)	-	0.431^a^	1 (33.3)	4 (17.4)	-	0.488^a^
		No	18 (52.9)	6 (75)			2 (66.7)	19 (82.6)		

Bold indicates *P* < 0.05.

a Fisher's exact test.

b Effect size analysis: Cramer's V (unordered variable).

c Extrusion disc: patients with free nucleus pulposus to the posterior border of the vertebral body after traumatic disc herniation(Figure 3); “-” Because the total number of cases analyzed was less than 40, Fisher's exact probability method was used.

### 3.4 Spinal cord function pre- and post-operation

Postoperatively, 26 patients exhibited no improvement in spinal cord function.By the final follow-up, 8 patients in the anterior group and 12 patients in the posterior group had died. The remaining patients with spinal cord injuries exhibited varying degrees of neurological recovery, with improvements ranging from 1 to 2 ASIA grades (Table 6).

**Table 6:** ASIA of spinal cord injuries at preoperative and final follow-up in both groups.

ASIA scale	Pre-op/n	Anterior group						Pre-op/n	Posterior group					
		A	B	C	D	E	Died		A	B	C	D	E	Died
A	10		2	2			6	12	2					10
B	2			2				6		2	1	2		1
C	11			7	3		1	5			1	3		1
D	57				11	45	1	22				3	19	
E	21					21		4					4	
Total	101		2	11	14	67	8	49	2	2	2	8	23	12

### 3.5 Radiographic parameters pre- and post-operation

Postoperatively, the cervical lordosis angle (CL), segmental Cobb angle (Lsa), and vertebral slippage distance (Sod) significantly improved compared to preoperative values (*P* < 0.05, Table 7). Both anterior and posterior groups, including their respective subgroups, showed comparable improvements in △CL, △Lsa, and △Sod, with no significant differences between them (*P* > 0.05, Table 8).

**Table 7:** Comparison of CL, Lsa, and Sod pre- and post-operation.

Imaging parameter	Pre-operation	Post-operation	*t*	*P*
CL (°)	−10.15 ± 12.08	−12.62 ± 9.72	3.15	**0**.**002**
Lsa (°)	2.29 ± 11.39	−4.83 ± 5.71	8.24	**<0**.**001**
Sod (mm)	4.66 ± 2.78	0.79 ± 1.09	15.87	**<0**.**001**

Bold indicates *P* < 0.05.

**Table 8:** Comparison of improvement in CL, Lsa, and Sod between inter-group and subgroups.

Imaging parameter		△CL (°)	△Lsa (°)	△Sod (mm)
Inter-group	Anterior	−3.51 ± 8.83	−8.08 ± 9.94	−3.54 ± 2.61
	Posterior	−0.31 ± 10.77	−5.12 ± 11.64	−4.53 ± 3.57
*t*		−1.94	−1.615	1.729
*p*		0.054	0.108	0.088
Anterior group	Single anterior	−3.62 ± 8.61	−7.78 ± 9.18	−3.47 ± 2.66
	Combined anterior-posterior	−2.64 ± 10.88	−10.55 ± 15.16	−4.09 ± 2.24
*t*		−0.348	0.871	0.740
*P*		0.729	0.386	0.461
Posterior group	Single posterior	1.38 ± 8.76	−2.69 ± 10.09	−3.86 ± 2.75
	Combined Posterior-anterior	−0.92 ± 11.46	−6.00 ± 12.16	−4.77 ± 3.82
*t*		0.656	0.876	0.784
*P*		0.515	0.385	0.437

### 3.6 Surgery-related complications

**Figure 6:**
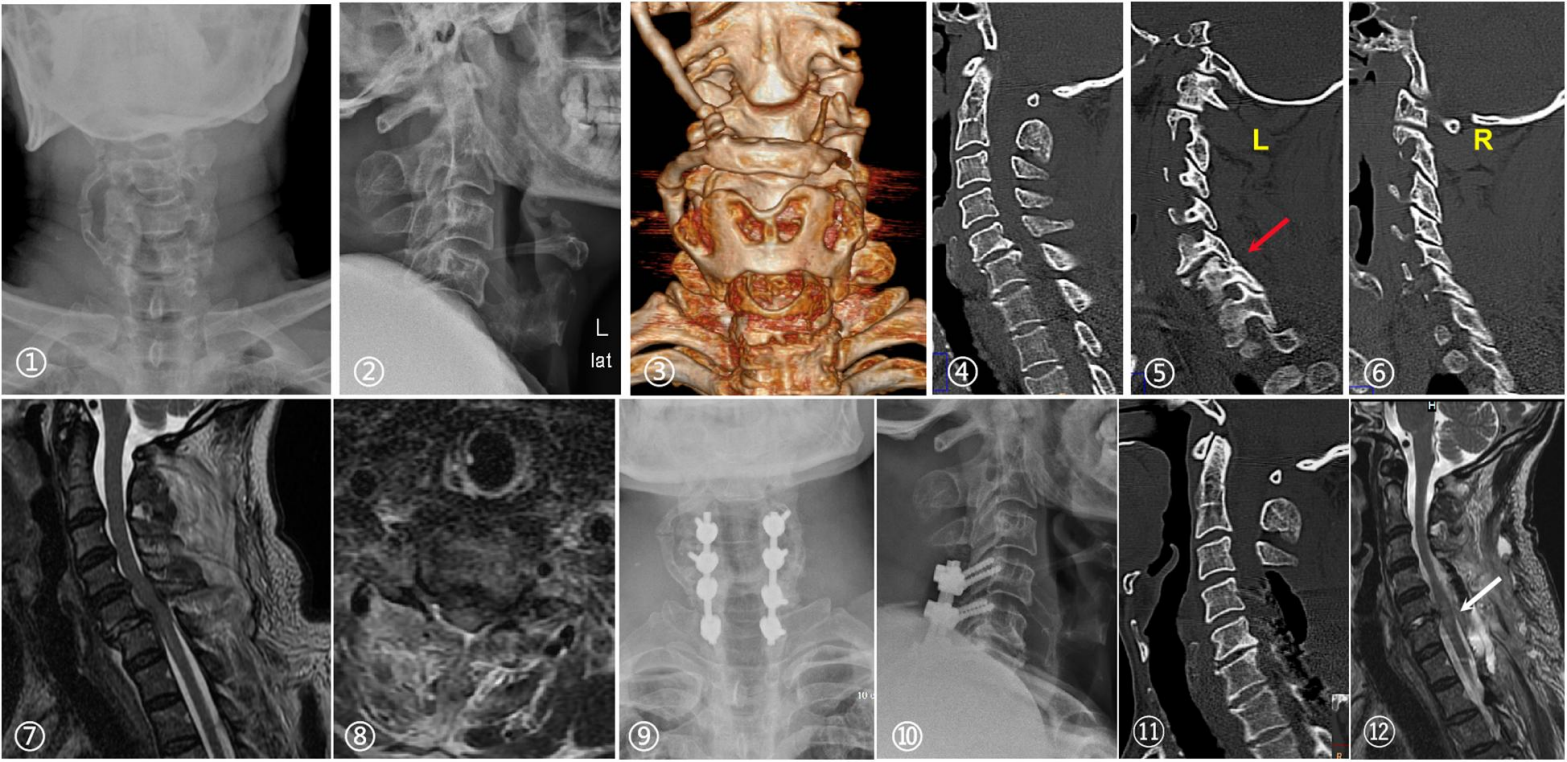
A 69-year-old male presented in July 2018 with “neck pain and restricted movement following a fall 12 h prior.” imaging confirmed a C5/6 fracture-dislocation with spinal cord injury (ASIA-D) and left facet dislocation with fracture [(2), (4), (5), (7), (8)]. An anterior approach was initially planned; however, bony fusion between the thyroid cartilage and hyoid bone [(1), (3)] prevented safe anterior retraction of the trachea and esophagus, necessitating a posterior approach. Preoperatively, the patient had largely recovered neurologically, so only facet joint unlocking, reduction, and posterior fixation were performed—without laminectomy or decompression. Following anesthesia recovery, the patient developed quadriplegia and decreased superficial sensation (ASIA-B). Emergency reoperation was performed with posterior laminectomy and extended decompression. Postoperative cervical x-rays [(9), (10)] and CT [(11)] confirmed C4–C6 laminectomy. MRI demonstrated signal changes in the spinal cord at C5/6 and confirmed adequate decompression [(12)].

A total of 10 patients (6.67%) experienced perioperative complications. In the anterior approach group, 3 patients (2.97%) developed complications. One patient required secondary posterior surgery due to poor facet alignment observed on follow-up CT after anterior-only surgery. Two cases of cerebrospinal fluid (CSF) leakage were managed with pressure drainage and intensified antibiotic therapy.In the posterior approach group, 7 patients (14.29%) developed complications, including two cases of deep incision infection. Three cases of CSF leakage were similarly managed without serious sequelae. Two cases of nerve damage, and one patient experienced neurological deterioration (from ASIA-D to ASIA-B) post-anesthesia and underwent emergency laminectomy, decompression, and posterior fixation; the patient improved to ASIA-C at discharge and achieved full recovery at final follow-up (Figure 6).

### 3.7 Surgical approach selection process

**Figure 7:**
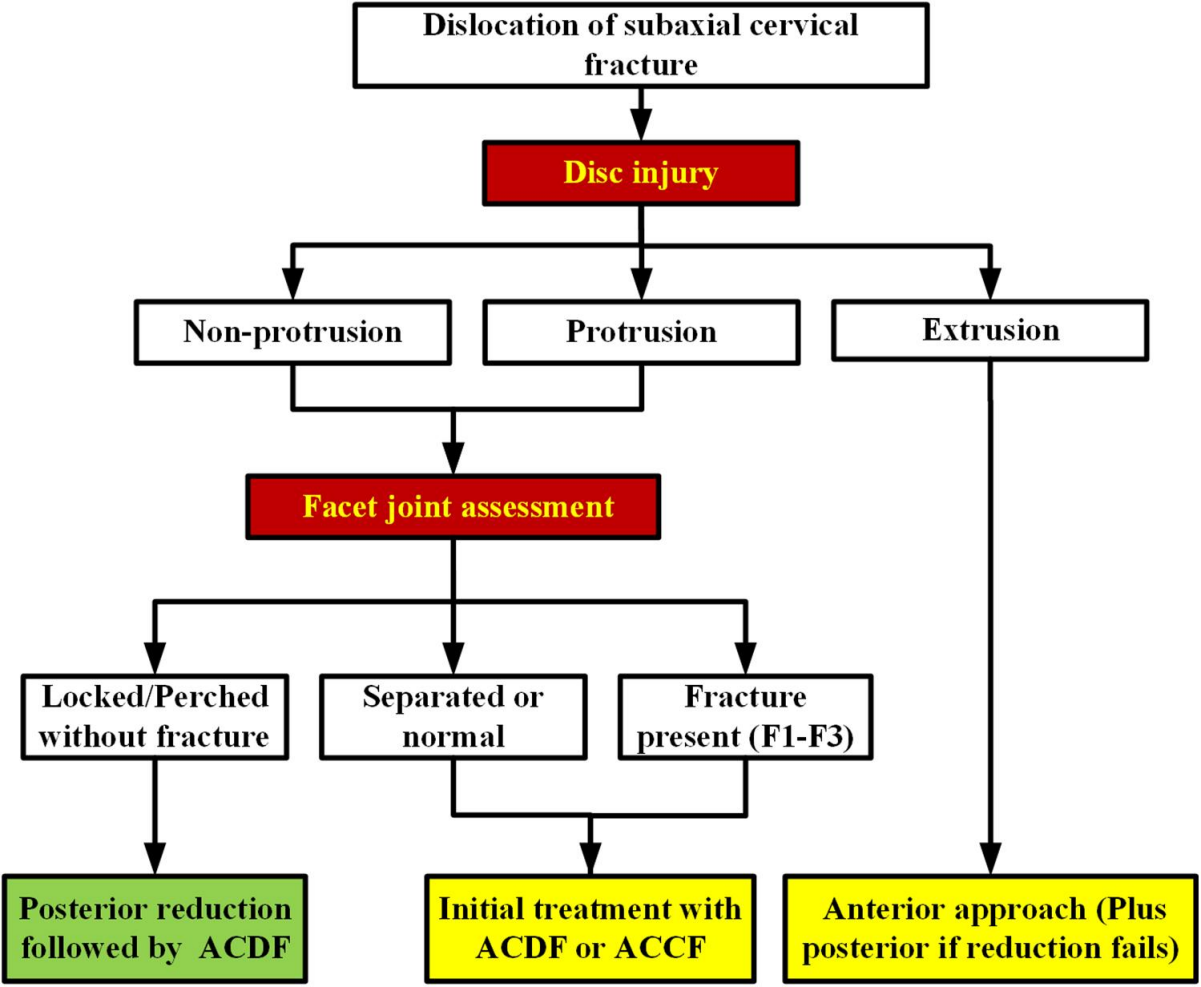
Illustrates the flowchart for determining the surgical approach to subaxial cervical spine fracture-dislocation.

In this study, intervertebral disc injuries at the dislocation segment were classified into three types: non-protrusion, protrusion, and extrusion. For extrusion-type injuries, an anterior approach is primarily recommended. If anterior reduction fails or posterior instability is observed intraoperatively, supplementary posterior fixation should be considered. For non-protrusion or protrusion-type injuries, further surgical planning is guided by facet joint evaluation: (1) In cases with locked or perched facet joints without associated fractures, posterior reduction followed by anterior cervical discectomy and fusion (ACDF) is advised. (2) If facet fractures are present (classified as F1-F3), an anterior approach such as ACDF or anterior cervical corpectomy and fusion (ACCF) is the preferred initial strategy. (3) When the facet joints are separated or appear normal, anterior treatment (ACDF or ACCF) is typically sufficient. This decision-making algorithm is illustrated in Figure 7.

## 4 Discussion

### 4.1 Characteristics of subaxial cervical fracture-dislocation

Subaxial cervical fracture-dislocation represents the most severe form of cervical spine injury, typically involving three-column disruption and malalignment caused by high-energy trauma. In our cohort, these injuries accounted for 34.5% (188/545) of subaxial cervical cases, with 83.3% (125/150) of surgically treated patients presenting with neurological deficits. The high incidence of neurological impairment underscores the vulnerability of the cervical spinal cord to mechanical compression and secondary injury mechanisms (1, 3, 4, 10, 15). The injury distribution showed predominance at C5–C7 levels (71.4%), consistent with this region's biomechanical susceptibility due to its transitional position between the mobile upper cervical spine and the relatively rigid thoracic spine. This anatomical characteristic, combined with the facet joint orientation and ligamentous attachments, makes this area particularly prone to dislocation injuries during high-energy trauma (1, 4).

### 4.2 The impact of disc injury on the surgical approach selection

Traumatic disc herniation in the context of subaxial cervical fracture-dislocation requires careful consideration during closed reduction and surgical management. In this study, preoperative MRI identified traumatic disc herniation in 46.7% of cases (70/150). For patients with posteriorly extruding discs, heavy traction was avoided, both preoperatively and intraoperatively. Instead, light traction (3 kg) was applied solely to maintain cervical stability. The anterior approach was preferred to remove the free disc fragment, followed by increased traction for distraction and reduction. If reduction failed, the incision was temporarily closed, and a posterior approach was used for unlocking, reduction, and fixation, followed by anterior fixation and fusion. This study highlights the importance of preoperative MRI to avoid posterior displacement of the herniated disc, which could result in irreversible spinal cord compression. Canseco et al. (8) reported that 74.1% of U.S. surgeons and 57.1% of European surgeons routinely perform MRI before intervention, even in patients without neurological symptoms.

Franz et al. (20) reviewed 197 cases and found that traumatic disc herniation is not an absolute contraindication for cranio-cervical traction or posterior open reduction, noting only one instance of neurological deterioration after traction. They emphasized that early spinal realignment may aid in neurological recovery, though the study quality was low and caution is advised (20). On the contrary, Ordonez et al. (21) advocated for anterior decompression, reduction, and stabilization to prevent catastrophic neurological complications from posterior open or closed reduction due to herniated discs, emphasizing safe decompression and facet joint reduction. They cautioned that posterior or closed reduction can worsen spinal cord injury when disc herniation is located behind the vertebral body (21). Berrington et al. (22)reported four cases of neurological deterioration following closed reduction, with MRI revealing herniated discs as the cause. They recommended preoperative MRI to prevent such complications (22). In our study, one patient undergoing posterior surgery experienced worsening neurological function (ASIA-D to ASIA-B) after open reduction without decompression. The patient underwent a second surgery for C4–6 laminectomy and fusion (Figure 6). Although this patient did not have significant disc herniation, osteophytes at the posterior margin of the dislocated segmental disc space severely reduced the spinal canal's effective space, increasing the risk of injury. This case underscores the importance of considering not only disc injuries but also pathological changes in the spinal canal, as closed or posterior open reduction could lead to catastrophic consequences.

For patients in whom anterior surgical options are limited, posterior reduction combined with laminectomy for decompression should be considered, with the extent of laminectomy determined by the degree of canal compression. In the posterior subgroup analysis, 46.9% of patients in the combined posterior-anterior group (23/49) had disc herniation, but none experienced postoperative neurological deterioration. This suggests that, with careful screening and risk control, the combination of anterior and posterior approaches may offer a viable option for disc herniation, achieving reduction, 360° fixation, and improved stability while avoiding extensive posterior fixation or repeated anterior-posterior-anterior surgeries.

### 4.3 The impact of facet injury on the surgical approach selection

The facet joint injuries in our series demonstrated distinct patterns that significantly influenced surgical decision-making: Unilateral injuries were predominantly associated with perched facets (69.8%) and F1/F2 fractures (81.0%), while bilateral injuries more frequently involved locked facets (40.2%) without fractures (62.1%). These patterns have important surgical implications, as they reflect different injury mechanisms and stability profiles (3, 4). The classification proposed by Tang et al. (23) provides a useful framework for understanding these injury patterns. Our experience confirms their observation that Type I injuries (no facet fracture) are particularly challenging to reduce through closed methods, often requiring posterior surgical approaches. Conversely, injuries with facet fractures (Types II and III) may be more amenable to anterior stabilization.

Neil et al. (3) suggested that in unilateral dislocations without fracture, the dislocated segment enters a pathologically stable state, with adjacent ligaments under high tension, making closed reduction more difficult. Similarly, Yang et al. (24) proposed a scoring system evaluating the bony and ligamentous components of the posterior column. Injuries scoring >7 were deemed highly unstable, warranting anterior-posterior combined fixation (24)—a view echoed by Thomas et al. (12) for Stage 3 and Stage 4 injuries, where supplementary posterior fixation is advised.

Our findings further emphasize the role of facet joint anatomy in surgical decision-making. Unilateral facet injuries were often associated with perched facets and F1/F2 fractures, while bilateral injuries showed more variability—including normal alignment, locked facets, separation, or no fracture (Table 2). On the affected side, locked or separated joints were more frequent in non-fracture cases, whereas perched facets often co-occurred with fractures (Table 3). Comparatively, anterior approach cases more often presented with unilateral injuries, perched facets, and fractures, while posterior approach cases were more commonly associated with locked but unfractured facets (Table 4). Based on these patterns, we propose that posterior reduction should be the first-line approach for perched or locked facets without fractures, provided there is no disc herniation. This simplifies the surgical plan and improves reduction success rates.

Special populations, such as those with ankylosing spondylitis (AS) or diffuse idiopathic skeletal hyperostosis (DISH), require individualized strategies due to altered biomechanics and increased fixation failure risk. Long-segment or circumferential constructs may be necessary in these cases.

### 4.4 Surgical approache for subaxial cervical spine fracture dislocation

#### 4.4.1 Anterior approach surgery

The anterior approach remains widely adopted for subaxial dislocation, particularly when disc herniation or facet fractures are present. In our series, 60% of cases were successfully treated with anterior-only surgery, avoiding the need for aggressive traction. Benefits included shorter operative times, reduced intraoperative blood loss, and fewer fused segments (5, 9–11, 14, 15). However, anterior fixation alone provides limited posterior column support and may be insufficient in cases with significant instability, especially in osteoporotic bone (10, 24). Cervical collars were used postoperatively for ≥3 months to ensure stabilization.

Patients without spinal cord compression (ASIA E) achieved excellent outcomes with anterior-only surgery. However, in cases of locked or perched facets without fractures, posterior approaches offered better reduction and stability.

#### 4.4.2 Posterior approach surgery

Though often considered a secondary option, the posterior approach is crucial in managing locked/perched facet dislocations without disc herniation. In our cohort, 32.7% (49/150) underwent posterior surgery as the initial approach, and an additional 10.9% (11/101) required posterior procedures after anterior surgery due to failed reduction or instability.

Wilhelmy et al. (2) reported a 100% reduction success rate with posterior surgery compared to 45% with anterior surgery in patients who failed closed reduction. Despite the higher complication rate observed in the posterior group (14.3% vs. 2.97%), this approach remains vital in cases with severe neurological deficits or anatomical barriers to anterior access.

Posterior decompression via laminectomy is particularly effective in ASIA A/B patients. Aarabi et al. (25) reported that sufficient decompression significantly improved neurological recovery (AIS improvement in 58.9% with full decompression vs. 18.5% with inadequate decompression). In our cohort, three-level laminectomy without ventral disc removal still achieved excellent outcomes in some patients. Overall, the posterior approach should be strongly considered in patients with locked facets, severe instability, or compromised anterior corridors, particularly when rapid decompression is critical.

Our findings complement and extend previous reports on surgical decision-making in subaxial cervical fracture-dislocation. Consistent with Ordonez et al. (21) and Tang et al. (23), this study confirms that locked facets without fracture are best managed through a posterior-first approach, while cases with facet fractures or disc extrusion favor anterior or combined anterior-posterior surgery. However, unlike earlier studies that focused mainly on single-path strategies, our data revealed that nearly one-third of patients required combined or staged procedures. This reflects the complex interplay between facet morphology, disc displacement, and reduction feasibility in real-world practice.

From a clinical standpoint, these findings underscore two major implications. First, preoperative imaging-based assessment of facet alignment and disc morphology is essential to prevent failed reduction or incomplete decompression, especially in patients with hidden disc extrusion. Second, overreliance on a single approach may lead to residual instability or neurological deterioration when posterior column disruption is underestimated. Therefore, surgeons should integrate CT-MRI findings within a structured decision-making algorithm to optimize surgical safety and stability. This integrative approach may minimize complications reported in earlier literature and enhance long-term functional outcomes.

### 4.5 The limitations and prospects of the study

The average time from injury to admission was over 40 h, with surgery delayed by an additional 5 days—factors that likely affected outcomes. These delays reflect systemic limitations common in developing regions, including referral inefficiencies and limited surgical availability. A study from Nepal reported similar challenges, with all patients treated >48 h after injury (26).

Despite these constraints, patients with complete cord injury (ASIA A) benefited from surgery. Early intervention remains ideal, but even delayed decompression and stabilization can improve neurological outcomes. A significant limitation is survivorship bias, especially in our neurological outcomes analysis. Survivors, with less severe injuries, may not represent the full range of injury severity, while those who died, due to trauma-related complications, likely had more severe injuries and worse outcomes, skewing our data. Therefore, the findings should be interpreted cautiously, as selection bias may have influenced neurological recovery. To address this, longer follow-up and prospective cohort studies are needed to assess long-term outcomes. And increase the assessment of postoperative functional improvement of patients to enhance the role of surgical options in improving patient recovery. Additionally, external validation of our decision-making model is necessary through a multicenter study.

Future integration of technologies such as artificial intelligence, 3D navigation, and anterior cervical pedicle screw systems may improve safety and accuracy for anterior-only stabilization in selected cases (6, 11). Additionally, developing a predictive algorithm based on injury morphology and imaging parameters could assist in surgical planning.

## 5 Conclusions

In subaxial cervical fracture-dislocation, the morphology of facet joint and disc injuries plays a decisive role in surgical approach selection. Anterior surgery is most appropriate for cases involving disc extrusion or facet fractures, whereas posterior approaches are better suited for locked or perched facets without fracture. Accurate preoperative assessment of injury patterns via CT and MRI allows for tailored surgical planning and may significantly improve neurological and functional outcomes.

## Data Availability

The original contributions presented in the study are included in the article/Supplementary Material, further inquiries can be directed to the corresponding authors.

## References

[1] Quarrington RD Jones CF Tcherveniakov P Clark JM Sandler SJI Lee YC Traumatic subaxial cervical facet subluxation and dislocation: epidemiology, radiographic analyses, and risk factors for spinal cord injury. Spine J: Off J N Am Spine Soc. (2018) 18:387–98. 10.1016/j.spinee.2017.07.175 28739474

[2] Wilhelmy B Serra R Patel P Stokum J Hasan O Zhao R Reduction of traumatic unilateral locked facet of the subaxial cervical spine: what predicts successful closed skeletal traction, and is anterior or posterior surgery superior after unsuccessful closed reduction? J Neurosurg Spine. (2025) 43:143–53. 10.3171/2025.3.SPINE241107 40408873

[3] Crawford NR Duggal N Chamberlain RH Park SC Sonntag VKH Dickman CA. Unilateral cervical facet dislocation: injury mechanism and biomechanical consequences. Spine. (2002) 27:1858–64. 10.1097/00007632-200209010-00010 12221349

[4] Nadeau M McLachlin SD Bailey SI Gurr KR Dunning CE Bailey CS. A biomechanical assessment of soft-tissue damage in the cervical spine following a unilateral facet injury. J Bone Joint Surg Am. (2012) 94:e156. 10.2106/JBJS.K.00694 23138243

[5] Donnarumma P Bozzini V Rizzi G Berardi A Merlicco G. Surgical management of C-type subaxial cervical fractures using cervical traction followed by anterior cervical discectomy and fusion within 12 h after the trauma. J Craniovertebral Junction Spine. (2017) 8:338–41. 10.4103/jcvjs.JCVJS_99_17 PMC5763591 29403246

[6] Liu K Zhang Z. A novel anterior-only surgical approach for reduction and fixation of cervical facet dislocation. World Neurosurg. (2019) 128:e362–9. 10.1016/j.wneu.2019.04.153 31029820

[7] Abdelgawaad AS Metry ABS Elnady B El Sheriff E. Anterior cervical reduction decompression fusion with plating for management of traumatic subaxial cervical spine dislocations. Glob Spine J. (2021) 11:312–20. 10.1177/2192568220903741 PMC8013942 32875864

[8] Canseco JA Schroeder GD Patel PD Grasso G Chang M Kandziora F Regional and experiential differences in surgeon preference for the treatment of cervical facet injuries: a case study survey with the AO spine cervical classification validation group. Eur Spine J. (2021) 30:517–23. 10.1007/s00586-020-06535-z 32700126

[9] Miao D-C Qi C Wang F Lu K Shen Y. Management of severe lower cervical facet dislocation without vertebral body fracture using skull traction and an anterior approach. Med Sci Monit. (2018) 24:1295–302. 10.12659/msm.908515 29500927 PMC5846369

[10] Jonayed S Choudhury AAM Alam MS Dastagir O. Efficacy, safety, and reliability of the single anterior approach for subaxial cervical spine dislocation. Cureus. (2023) 15:e34787. 10.7759/cureus.34787 36777970 PMC9909243

[11] Liu K Zhang Z. Reduction of lower cervical facet dislocation: a review of all techniques. Neurospine. (2023) 20:181–204. 10.14245/ns.2244852.426 37016866 PMC10080426

[12] Henriques T Olerud C Bergman A Jónsson H. Distractive flexion injuries of the subaxial cervical spine treated with anterior plate alone. J Spinal Disord Tech. (2004) 17:1–7. 10.1097/00024720-200402000-00002 14734968

[13] Singh A El-Hajj VG Fletcher-Sandersjöö A Aziz N Ghaith AK Tatter C Predictors of failure after primary anterior cervical discectomy and fusion for subaxial traumatic spine injuries. Eur Spine J. (2024) 33:2332–9. 10.1007/s00586-024-08264-z 38664273

[14] Ren C Qin R Wang P Wang P. Comparison of anterior and posterior approaches for treatment of traumatic cervical dislocation combined with spinal cord injury: minimum 10-year follow-up. Sci Rep. (2020) 10:10346. 10.1038/s41598-020-67265-2 32587305 PMC7316727

[15] Botelho RV de Freitas Bertolini E Barcelos ACES Walter Daniel J Fernandes Joaquim A Dantas FLR The surgical treatment of subaxial acute cervical spine facet dislocations in adults: a systematic review and meta-analysis. Neurosurg Rev. (2022) 45:2659–69. 10.1007/s10143-022-01808-1 35596874

[16] Leng A Meng L Li J Shi S Guo M Yu H MRI-based surgical planning for irreducible subaxial cervical fracture-dislocation with bilateral locked facet joints: a retrospective cohort study. Orthop Surg. (2025) 17:1844–51. 10.1111/os.70054 40309846 PMC12146142

[17] Vaccaro AR Koerner JD Radcliff KE Oner FC Reinhold M Schnake KJ AOSpine subaxial cervical spine injury classification system. Eur Spine J. (2016) 25:2173–84. 10.1007/s00586-015-3831-3 25716661

[18] Roberts TT Leonard GR Cepela DJ. Classifications in brief: American spinal injury association (ASIA) impairment scale. Clin Orthop Relat Res. (2017) 475:1499–504. 10.1007/s11999-016-5133-4 27815685 PMC5384910

[19] Riew KD Yang JJ Chang D-G Park S-M Yeom JS Lee JS What is the most accurate radiographic criterion to determine anterior cervical fusion? Spine J. (2019) 19:469–75. 10.1016/j.spinee.2018.07.003 29990594

[20] Onishi FJ Daniel JW Joaquim AF Evangelista AC de Freitas Bertolini E Dantas FR The impact of traumatic herniated discs in cervical facets dislocations treatments: systematic review and meta-analysis. Eur Spine J. (2022) 31:2664–74. 10.1007/s00586-022-07290-z 35763222

[21] Ordonez BJ Benzel EC Naderi S Weller SJ. Cervical facet dislocation: techniques for ventral reduction and stabilization. J Neurosurg. (2000) 92:18–23. 10.3171/spi.2000.92.1.0018 10616053

[22] Berrington NR van Staden JF Willers JG van der Westhuizen J. Cervical intervertebral disc prolapse associated with traumatic facet dislocations. Surg Neurol. (1993) 40:395–9. 10.1016/0090-3019(93)90219-q 8211656

[23] Tang C Fan YH Liao YH Tang Q Ma F Wang Q Classification of unilateral cervical locked facet with or without lateral mass-facet fractures and a retrospective observational study of 55 cases. Sci Rep. (2021) 11:16615. 10.1038/s41598-021-96090-4 34400738 PMC8367956

[24] Yang J-S Liu P Liu T-J Zhang H-P Zhang Z-P Yan L When is the circumferential stabilization necessary for subaxial cervical fracture dislocations? The posterior ligament-bone injury classification and severity score: a novel treatment algorithm. Eur Spine J. (2021) 30:524–33. 10.1007/s00586-020-06580-8 32876731

[25] Aarabi B Olexa J Chryssikos T Galvagno SM Hersh DS Wessell A Extent of spinal cord decompression in motor complete (American spinal injury association impairment scale grades a and B) traumatic spinal cord injury patients: post-operative magnetic resonance imaging analysis of standard operative approaches. J Neurotrauma. (2019) 36:862–76. 10.1089/neu.2018.5834 30215287 PMC6484360

[26] Dhakal GR Bhandari R Dhungana S Poudel S Gurung G Kawaguchi Y Review of subaxial cervical spine injuries presenting to a tertiary-level hospital in Nepal: challenges in surgical management in a third world scenario. Glob Spine J. (2019) 9:713–6. 10.1177/2192568219833049 PMC6745644 31552151

